# Mastering fate: Redistributing a pioneer protein to rewrite leukemia's script

**DOI:** 10.1002/hem3.70084

**Published:** 2025-01-22

**Authors:** Yizhou Huang, Charles E. de Bock

**Affiliations:** ^1^ Children's Cancer Institute, Lowy Cancer Research Centre Randwick NSW Australia; ^2^ School of Clinical Medicine, Faculty of Medicine UNSW Sydney Sydney NSW Australia

The pioneer transcription factor PU.1 plays a crucial role in hematopoiesis, particularly during myeloid and lymphoid differentiation. PU.1 dysregulation has been implicated in leukemia development, including acute myeloid leukemia (AML) and acute lymphoblastic leukemia (ALL). However, developing therapeutic agents that directly target transcription factors has been challenging. This is partly due to the lack of well‐defined active sites amenable to pharmacological inhibition, making them undruggable and indispensable functions in healthy cells.^1^

In a new study led by Samuel Taylor in the laboratory of Ulrich Steidl, the authors took another approach to block PU.1 activity using a small molecule inhibitor that specifically blocked PU.1's interaction with DNA. Interestingly, rather than a global loss of PU.1 binding, the authors found that PU.1 was redistributed to alternative DNA sites within the genome, activating cellular differentiation.^2^ The implications of this therapeutic concept, particularly in relation to differentiation therapy, represent an important new development for the treatment of leukemia.

## PU.1 REDISTRIBUTION: UNRAVELING THE MOLECULAR BIOLOGY

**Figure 1:**
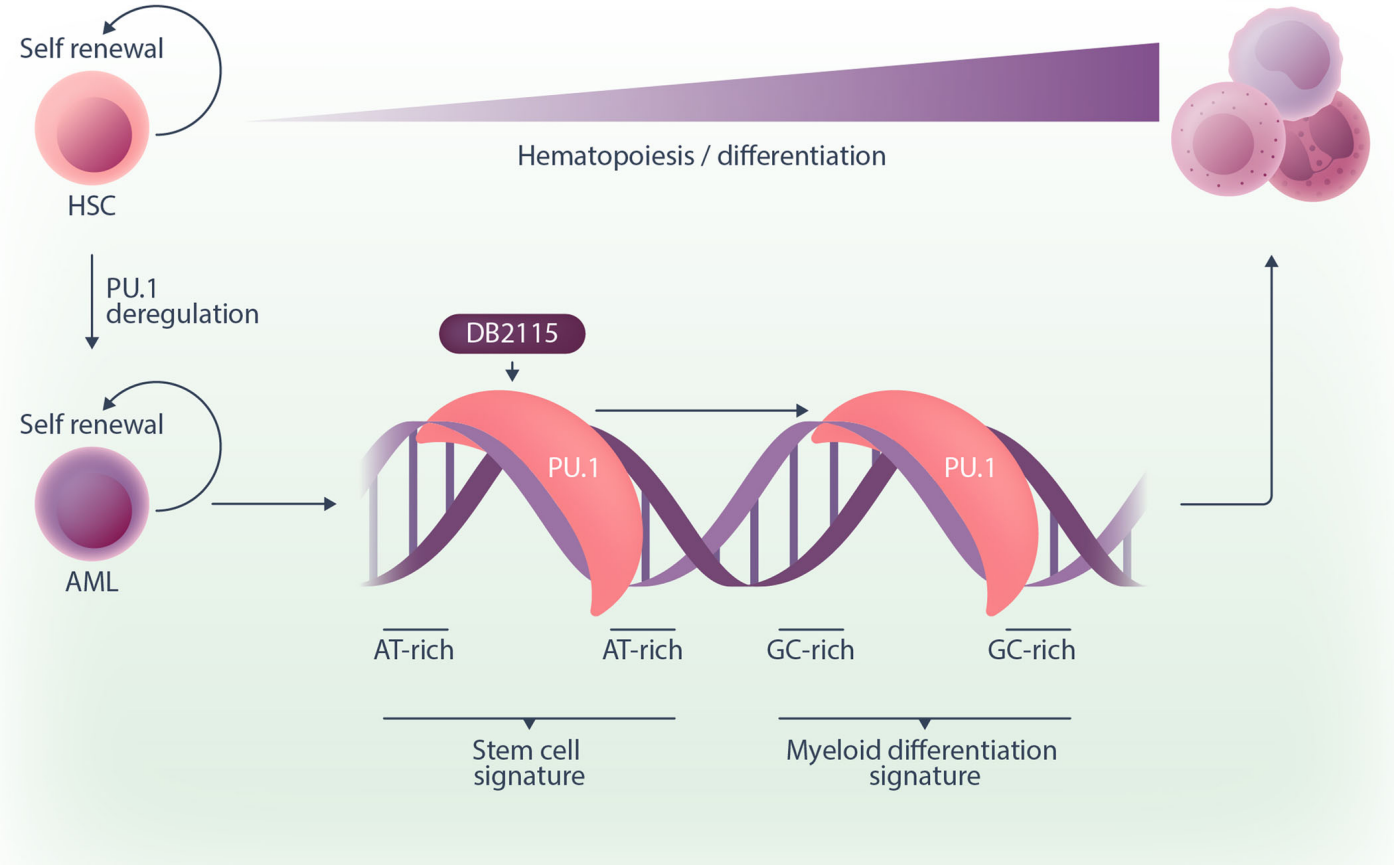
**Small molecule‐induced PU.1 redistribution drives AML differentiation.** PU.1 plays a crucial role in hematopoiesis, while its dysregulation occurs in 50% of AML samples and drives aberrant cellular proliferation and survival. A new small molecule inhibitor DB2115 targets and prevents PU.1 binding at consensus motif sites flanked by AT‐rich sequences. This results in a fraction of PU.1 being redistributed to alternative genomic regions flanked by GC‐rich sequences. This PU.1 redistribution drives a cell differentiation transcriptional program, providing a new therapeutic avenue for AML patients. AML, acute myeloid leukemia; AT, adenine–thymine; GC, guanine–cytosine.

Unlike kinases or other enzymes, transcription factors lack catalytic domains that can be inhibited, making direct pharmacological intervention more challenging. The new agents used by Taylor et al. are DNA‐binding heterocyclic diamidines, designed to specifically bind adenine–thymine (AT)‐rich minor grooves at DNA sites targeted by PU.1, thereby inhibiting PU.1 binding. Treatment of AML cells with this class of agents (DB2115, DB2373, DB2826, and DB2313) resulted in a significant decrease in AML cell proliferation. Originally, the authors hypothesized that this was a consequence of drug‐induced global loss of PU.1 binding across the genome. Surprisingly, they then found that not only did the vast majority of PU.1 sites remain unchanged after drug treatment, but there was nearly an equivalent loss and gain of PU.1 binding sites. Their data instead support a model in which PU.1 is redistributed to alternative genomic regions rather than global loss of binding (Figure 1).

To understand the molecular biology underlying this observation, the authors used numerous elegant sequencing techniques such as CLICK‐on‐CUT&Tag, ATAC‐sequencing, and ChIP‐sequencing temporally to directly show where the drug bound, PU.1 was lost and chromatin then closed. Conversely, closed chromatin regions that gained PU.1 binding were remodeled to become more accessible. Using transcriptomics and gene set enrichment analysis, the authors showed that these gained PU.1‐bound regions were involved in “cellular differentiation,” whereas the PU.1‐lost regions were enriched for a “stem cell signature” (Figure 1). Hence, these drugs pharmacologically reprogrammed PU.1‐mediated transcriptional activity to drive cellular differentiation instead of completely suppressing its activity. This represents a significant shift in our thinking about targeted therapies for AML; rather than focusing on cell death as the ultimate endpoint, here the PU.1 redistributor drug works by nudging the leukemia cells toward normal terminal differentiation where cells lose proliferative potential, fundamentally altering the malignant phenotype.

Similar to PU.1, HOXA9 is another key transcription factor implicated in the maintenance of leukemic stem cells and plays a critical role in AML pathogenesis. Like the work by Taylor et al., a recent independent study also used heterocyclic diamidines to inhibit HOXA9 binding to DNA in AML, resulting in strong anti‐leukemic effects and AML cell differentiation with minimal impact on normal hematopoiesis.^3^ Given this new work by Taylor et al., it would be interesting to determine whether this HOXA9 inhibition‐induced anti‐leukemic action is also due to HOXA9 redistribution to alternative sites within the genome.

## EXPANDING DIFFERENTIATION THERAPY: CHALLENGES AND FUTURE DIRECTIONS

The concept of differentiation therapy is not new, with its success well‐documented in the treatment of acute promyelocytic leukemia (APL) with all‐trans retinoic acid (ATRA). In APL, the fusion oncoprotein PML::RARA blocks the differentiation of promyelocytes into mature granulocytes. ATRA acts by dissociating the transcriptional co‐repressor complex bound by PML::RARA, allowing for the restoration of normal gene transcription and cellular differentiation.^4^ Remarkably, this differentiation therapy has led to high cure rates without the need for traditional cytotoxic chemotherapy.

While the results showing the agents disrupting transcription factor–DNA interactions are promising, several challenges remain. First, the translation of this approach from bench to bedside will require extensive preclinical and clinical testing. In particular, studies in combination with either standard‐of‐care or novel targeted agents (e.g., the menin inhibitor *revumenib*, which was FDA‐approved in November 2024) are warranted in the future to achieve optimal patient outcomes.^5^ Additionally, with 50% of AML samples having deregulated PU.1,^6^ it is unclear whether only these patients will respond to such treatment. To this end, Taylor et al. showed that treating seven primary AML samples resulted in reduced cell growth and colony formation in vitro, but its utility across different AML subtypes is yet to be determined. Finally, the potential for acquired resistance must be considered especially in light of the genetic and epigenetic heterogeneity present in AML patients. In APL, resistance to ATRA has been documented, typically due to mutations in the PML::RARA fusion protein.^4^ Whether similar resistance mechanisms could emerge with PU.1 redistribution is an open question and one that future research will need to address. Nevertheless, the exploration of pharmacologically driven PU.1 redistribution represents an exciting advancement in AML therapy.

## CONCLUDING REMARKS

Ectopic or deregulated transcription factor expression is a hallmark of AML, ALL, and many other cancers. The discovery of drug‐induced transcription factor redistribution provides a new treatment paradigm to reprogram malignant cells toward terminal differentiation across diverse cancer types.

## AUTHOR CONTRIBUTIONS

Both Yizhou Huang and Charles E. de Bock conceptualized and co‐wrote the article. Both authors agreed to the final version.

## CONFLICT OF INTEREST STATEMENT

The authors declare no conflicts of interest.

## FUNDING

No funding was received for this publication.

## Data Availability

Data sharing is not applicable to this article as no data sets were generated or analyzed during the current study.
